# Hydrodynamics of a Flexible Soft-Rayed Caudal Fin

**DOI:** 10.1371/journal.pone.0163517

**Published:** 2016-10-03

**Authors:** Gil Iosilevskii

**Affiliations:** Faculty of Aerospace Engineering, Technion, Haifa, Israel; Universitat Zurich, SWITZERLAND

## Abstract

The paper addresses hydrodynamic performance of a slender swimmer furnished with a flexible small-aspect-ratio soft-rayed caudal fin. The recoil of the fin is found by solving the coupled hydro-elastic problem, in which the structure of the fin is modeled by a cantilever of variable cross section and the hydrodynamic forces acting on it are modeled using the elongated body theory. It is shown that the recoil has practically no effect on the propulsion efficiency of anguilliform swimmers, but has a profound effect on the efficiency of carangiform swimmers, which can increase almost four-fold between low-speed (low-thrust) cruise and high-speed (high-thrust) burst. Whilst the magnitude of this effect furnishes a plausible argument in favor of burst-and-coast locomotion strategies, it also infers that carangiform swimmers cannot rely on elastic recoil of the caudal fin to be efficient throughout the usable speed range, and must actively flex it at low speeds.

## 1. Introduction

In order to swim efficiently using body-and-caudal-fin (BCF) propulsion, the caudal fin has to flex in coordination with its lateral motion, turning left when moving right and vice versa. This conjecture is actually inferred by the elongated body theory [1–3]–and it will be plainly recapitulated in Section 4.1 below–but the fact is that the caudal fin does flex in all BCF swimmers.

The flex of the caudal fin can be active, reflecting the action of caudal muscles [4,5], or passive, reflecting the elastic deformation of the fin under hydrodynamic loads [6,7]. Although the caudal muscles are indisputably active during slow swimming [4,5], whether the flex of caudal fin is active or passive at all swimming speeds is still debatable [8]–furnishing an answer to this question is one of the objectives of this study. It will be done by *reductio ad absurdum*; that is, by accepting the hypothesis that the flex of the fin is passive and assessing its consequences. Performance of a BCF swimmer furnished with a flexible caudal fin is another objective of this study.

Because the thrust generated by the swimmer and the power needed to this end are periodic, swimming performance is conveniently assessed using time-averaged quantities: speed, thrust, power, etc. The averaging period can be as short as a single tail-beat or as long as numerous tail-beats. The single-tail-beat averaged performance can be expressed in terms of propulsion efficiency: the ratio between the power made good (the product of thrust and speed) and the power actually spent. It is determined mainly by the morphology of the swimmer and its swimming gait; as such, it is affected by flexibility of the caudal fin. The long-term averaged performance can be expressed in terms of locomotion efficiency: the ratio between the energy needed to drag the swimmer between the beginning and end of the course at the average swimming speed, and the energy actually spent. It reflects the effective propulsion efficiency of the swimmer along the course. Propulsion efficiency is addressed herein; locomotion efficiency is addressed in the companion paper [9].

The single-tail-beat-averaged performance can be evaluated only if the shape of the swimmer–and, in particular, the shape of its caudal fin–is known throughout the averaging period. The shape of a passively flexing fin is determined by the balance between elastic, hydrodynamic and inertial forces. Preferring simplicity to accuracy, the elastic forces will be found by representing the fin as an equivalent cantilever [10], whereas the hydrodynamic forces will be found using the elongated body theory. Comparable combination has been used in Ref. [11] for the study of a flexible slender propulsor.

## 2. Preliminaries

**Fig 1:**
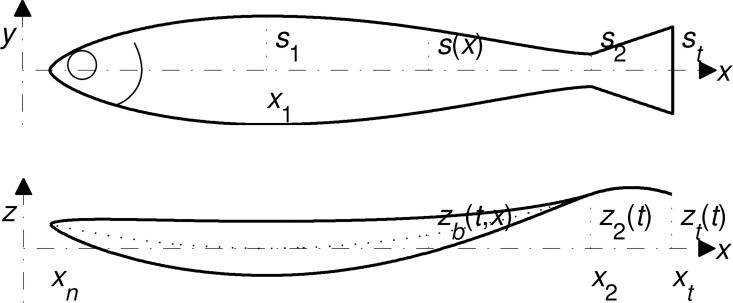
The reference frame and the notation.

Consider a fish, swimming at constant speed *v* along a straight path–its body bending left and right relative to that path. A right-handed reference frame will follow the fish as it swims, the *x*- and *y*-axes pointing backwards, along the swimming path, and upwards, parallel to the flat side of the caudal fin (Fig 1). The unbent fish will be assumed symmetrical with respect to both the *x-z* (coronal) and the *x-y* (sagittal) planes. The projection of the fish, body and fins together, onto the *x-y* plane starts with a point at *x* = *x*_*n*_, reaches the maximal span 2*s*_1_ at *x* = *x*_1_, and narrows back to 2*s*_2_ at *x* = *x*_2_, the caudal peduncle. The caudal fin starts with the span 2*s*_2_ at *x* = *x*_2_, and reaches the maximal span 2*s*_*t*_ at *x* = *x*_*t*_, the posterior end; the trailing edge of the fin is straight. In between, the local semi-span of the fish is described by a real-valued function *s* on (*x*_*n*_,*x*_*t*_); in particular, *s*(*x*_*n*_) = 0, *s*(*x*_1_) = *s*_1_, *s*(*x*_2_) = *s*_2_, and *s*(*x*_*t*_) = *s*_*t*_. *s*_1_ and *s*_*t*_ are small as compared with the length of the fish, *l* = *x*_*t*_–*x*_*n*_; *ds*/*dx* is small as compared with unity on the widening segments, (*x*_*n*_,*x*_1_) and (*x*_2_,*x*_*t*_).

It will be assumed that cross-sections of the fish, caudal fin included, do not distort during swimming; moreover, the caudal fin is vanishingly thin. The lateral displacement of the mid-plane of the fish from the *x-y* plane will be described by the real-valued function *z*_*b*_, defined on (−∞,∞) × (*x*_*n*_,*x*_*t*_). The derivative of *z*_*b*_,

$$\alpha_{b}\left(t,x\right)=\partial z_{b}\left(t,x\right)/\partial x,$$ (1)

will be assumed small for every time *t* and every *x* ∈ (*x*_*n*_,*x*_*t*_); it can be interpreted as the angle between the mid-plane of the fish and the swimming direction. *z*_*b*_ and *α*_*b*_ are known on (−∞,∞) × (*x*_*n*_,*x*_2_), but not on (−∞,∞) × (*x*_2_,*x*_*t*_). Given that *α*_*b*_ is small, the origin of the reference frame can be adjusted to have

$$x_{2}=0.$$ (2)

The left-right flex of the fin,

$$z_{f}\left(t,x\right)=z_{b}\left(t,x\right)-z_{b}^{\prime }\left(t,x\right),$$ (3)

will be defined as the lateral displacement of the fin from the linear continuation of the mid plane of the body beyond the caudal peduncle,

$$z_{b}^{\prime }\left(t,x\right)=z_{b}\left(t,x_{2}\right)+\alpha_{b}\left(t,x_{2}\right)x.$$ (4)

*z*_*b*_(*t*,*x*_2_) and *α*_*b*_(*t*,*x*_2_) will be abbreviated as *z*_2_ (*t*) and *α*_2_ (*t*); likewise, *z*_*b*_(*t*,*x*_*t*_) and *α*_*b*_(*t*,*x*_*t*_) will be abbreviated by *z*_*t*_(*t*) and *α*_*t*_(*t*).

## 3. Flex of the Caudal Fin

Given that cross sections of the fin do not deform when the fin flexes left-right, we model the fin as a cantilever, rigidly attached to the fish body at the caudal peduncle. Consequently, the instantaneous flex of the fin can be found as the solution of differential equation

$$\frac{\partial^{2}}{\partial x^{2}}\left(EI\left(x\right)\frac{\partial^{2}z_{f}\left(t,x\right)}{\partial x^{2}}\right)=f_{⊥}\left(t,x\right)-m\left(x\right)\frac{\partial^{2}z_{b}\left(t,x\right)}{\partial t^{2}},$$ (5)

subject to edge conditions,

$$\frac{\partial }{\partial x}I\left(x\right)\frac{\partial^{2}z_{f}\left(t,x\right)}{\partial x^{2}}=0\;\mathrm{and}\;I\left(x\right)\frac{\partial^{2}z_{f}\left(t,x\right)}{\partial x^{2}}=0\;\mathrm{at}\;x=x_{t},$$ (6)

$$\partial z_{f}\left(t,x\right)/\partial x=0\;\mathrm{and}\;z_{f}\left(t,x\right)=0\;\mathrm{at}\;x=x_{2}.$$ (7)

Here, *E* is the effective Young’s modulus of the fin’s structural skeleton, *I* is the area moment of the skeleton’s cross section, *f*_⊥_ is the lateral component of the hydrodynamic force per unit length acting on the fin, and *m* is the mass of the fin per unit length. The reader is referred to Ref. [10] for details. The contribution of the in-plane component of the hydrodynamic force, *f*_∥_, has been tacitly neglected; the conditions under which this assumption is coherent are discussed in Appendix A.

Under the present set of assumptions, the hydrodynamic loads acting on the caudal fin can be effectively found in the framework of the elongated body theory [1–3,12,13]. Notwithstanding its simplicity, the practical implementation of this theory for analysis of a swimming fish is hindered by the sheer number of free parameters. In fact, the hydrodynamic loads on the fin depend on not only the shape and motion of the fin itself, but also on the shape and motion of the fish body anteriad of it–see equations (S1)-(S8) in S1 File. This dependence manifests the hydrodynamic interaction between the caudal fin and the wake, released from the dorsal and ventral edges of the converging segment of the fish, (*x*_1_,*x*_2_). If the vortices comprising this wake were weak–as could have happened if the lateral displacement of the deepest section of the fish were small–the loads on the caudal fin could have been assumed independent of the shape of the fish. Following Ref. [2], and invoking the same arguments in favor of simplicity that were invoked thereat, this case will be the one assumed below; it is shown in S1 File, that it represents a formal leading order approximation with respect to the lateral displacement of the deepest section of the fish. Under these assumptions,

$$f_{⊥}\left(t,x\right)=-\pi \rho \frac{D}{Dt}\left(s^{2}\left(x\right)\frac{Dz_{b}\left(t,x\right)}{Dt}\right)$$ (8)

for each *x* ∈ (*x*_2_,*x*_*t*_). Here, *D*/*Dt* = ∂/∂*t* + *v*∂/∂*x* is the convective derivative and *ρ* is the density of water. This is Eq (16) in Ref. [12]; its derivation can be found in S1 File; the reader is referred to the paragraph immediately following (S8). Introducing *z*_*b*_ from (3), it takes on the explicit form

$$\begin{array}{l} f_{⊥}\left(t,x\right)=-\pi \rho v\frac{ds^{2}}{dx}\left(\frac{\partial z_{b}}{\partial t}+v\frac{\partial z_{b}}{\partial x}\right)-\pi \rho s^{2}\left(\frac{\partial^{2}z_{b}}{\partial t^{2}}+2v\frac{\partial^{2}z_{b}}{\partial t\partial x}+v^{2}\frac{\partial^{2}z_{b}}{\partial x^{2}}\right)\\ \;=-\pi \rho v^{2}\frac{ds^{2}}{dx}\left(\frac{1}{v}\frac{\partial z_{f}}{\partial t}+\frac{\partial z_{f}}{\partial x}\right)-\pi \rho v^{2}s^{2}\left(\frac{1}{v^{2}}\frac{\partial^{2}z_{f}}{\partial t^{2}}+\frac{2}{v}\frac{\partial^{2}z_{f}}{\partial t\partial x}+\frac{\partial^{2}z_{f}}{\partial x^{2}}\right)\\ \;-\pi \rho v^{2}\frac{ds^{2}}{dx}\left(\frac{1}{v}\frac{dz_{2}}{dt}+\frac{x}{v}\frac{d\alpha_{2}}{dt}+\alpha_{2}\right)-\pi \rho v^{2}s^{2}\left(\frac{1}{v^{2}}\frac{d^{2}z_{2}}{dt^{2}}+\frac{x}{v^{2}}\frac{d^{2}\alpha_{2}}{dt^{2}}+\frac{2}{v}\frac{d\alpha_{2}}{dt}\right), \end{array}$$ (9)

where the arguments, *t* and *x* of *z*_*b*_ and *z*_*f*_, *x* of *s*, and *t* of *z*_2_ and *α*_2_, have been omitted for brevity.

By interpretation, the coefficient *πρs*^2^ with ∂^2^*z*_*b*_/∂^2^*t*, ∂^2^*z*_*f*_/∂^2^*t* and ∂^2^*z*_2_/∂^2^*t* is the added mass of the fin per unit length [2]. The physical mass of the fin per unit length is *ρ*_*f*_2*sθ*, where *ρ*_*f*_ and *θ* are the effective density and thickness of the fin. Because the density of the fin is almost the same as that of water, and because the thickness of the fin is invariably small when compared with its span, the mass of the fin turns negligible when compared with its added mass. Consequently, the last term on the right-hand side of (5) will be neglected hereafter.

Substituting (9) for the remaining term, Eq (5) will be recast in dimensionless form:

$$\begin{array}{l} \bar{\kappa }\frac{\partial^{2}}{\partial {\bar{x}}^{2}}\left(\bar{I}\frac{\partial^{2}{\bar{z}}_{f}}{\partial {\bar{x}}^{2}}\right)+\frac{d{\bar{s}}^{2}}{d\bar{x}}\left(\frac{\partial {\bar{z}}_{f}}{\partial \bar{t}}+\frac{\partial {\bar{z}}_{f}}{\partial \bar{x}}\right)+{\bar{s}}^{2}\left(\frac{\partial^{2}{\bar{z}}_{f}}{\partial {\bar{t}}^{2}}+2\frac{\partial^{2}{\bar{z}}_{f}}{\partial \bar{t}\partial \bar{x}}+\frac{\partial^{2}{\bar{z}}_{f}}{\partial {\bar{x}}^{2}}\right)\\ \;=-\frac{d{\bar{s}}^{2}}{d\bar{x}}\frac{d{\bar{z}}_{2}}{d\bar{t}}-{\bar{s}}^{2}\frac{d^{2}{\bar{z}}_{2}}{d{\bar{t}}^{2}}-\frac{d{\bar{s}}^{2}}{d\bar{x}}\left(\frac{d{\bar{\alpha }}_{2}}{d\bar{t}}\bar{x}+{\bar{\alpha }}_{2}\right)-{\bar{s}}^{2}\left(\frac{d^{2}{\bar{\alpha }}_{2}}{d{\bar{t}}^{2}}\bar{x}+2\frac{d{\bar{\alpha }}_{2}}{d\bar{t}}\right), \end{array}$$ (10)

where,

$$\bar{x}=x/l_{t},\;\bar{t}=tv/l_{t},$$ (11)

$$\bar{\kappa }=\frac{E}{\rho v^{2}}\frac{I\left(0\right)}{\pi s_{t}^{2}l_{t}^{2}},$$ (12)

$${\bar{z}}_{b}\left(\bar{t},\bar{x}\right)=z_{b}\left(t,x\right)/l_{t},\;{\bar{z}}_{f}\left(\bar{t},\bar{x}\right)=z_{f}\left(t,x\right)/l_{t},\;{\bar{z}}_{2}\left(\bar{t}\right)=z_{2}\left(t\right)/l_{t},\;{\bar{\alpha }}_{2}\left(\bar{t}\right)=\alpha_{2}\left(t\right),$$ (13)

$$\bar{s}\left(\bar{x}\right)=s\left(x\right)/s_{t},$$ (14)

$$\bar{I}\left(\bar{x}\right)=I\left(x\right)/I\left(0\right),$$ (15)

and in which *l*_*t*_ = *x*_*t*_–*x*_2_ is the length of the caudal fin. $\bar{\kappa }$ will be interpreted as the reduced stiffness of the fin (note that it depends on the swimming speed).

Because Eq (10) and its edge conditions, (6) and (7), are linear, substituting

z¯2(t¯)=Re(z^2eiω¯t¯)andα¯2(t¯)=Re(α^2eiω¯t¯), (16)

implies

z¯f(t¯,x¯)=Re(z^f(x¯)eiω¯t¯), (17)

and, in particular,

z¯t(t¯)=Re(z^teiω¯t¯)andα¯t(t¯)=α¯2(t¯)+(∂z¯f/∂x¯)x¯=x¯t=Re(α^teiω¯t¯). (18)

In (16–18), an over-hat denotes a complex-valued amplitude, whereas $\bar{\omega }$ denotes the reduced frequency, related with the ‘real’ frequency, *ω*, by

$$\bar{\omega }=\omega l_{t}/v.$$ (19)

Substituting (16) and (17) for ${\bar{z}}_{2}$, ${\bar{\alpha }}_{2}$ and ${\bar{z}}_{f}$, Eq (10) becomes

$$\begin{array}{l} \bar{\kappa }\frac{d^{2}}{d{\bar{x}}^{2}}\left(\bar{I}\frac{d^{2}{\hat{z}}_{f}}{d{\bar{x}}^{2}}\right)+\frac{d}{d\bar{x}}\left({\bar{s}}^{2}\frac{d{\hat{z}}_{f}}{d\bar{x}}\right)+i\bar{\omega }\left(\frac{d}{d\bar{x}}\left({\bar{s}}^{2}{\hat{z}}_{f}\right)+{\bar{s}}^{2}\frac{d{\hat{z}}_{f}}{d\bar{x}}\right)+{\left(i\bar{\omega }\right)}^{2}{\bar{s}}^{2}{\hat{z}}_{f}\\ \;=-\left(\frac{d{\bar{s}}^{2}}{d\bar{x}}+i\bar{\omega }{\bar{s}}^{2}\right)i\bar{\omega }{\hat{z}}_{2}-\left(\frac{d{\bar{s}}^{2}}{d\bar{x}}+i\bar{\omega }\left(\bar{x}\frac{d{\bar{s}}^{2}}{d\bar{x}}+2{\bar{s}}^{2}\right)+{\left(i\bar{\omega }\right)}^{2}\bar{x}{\bar{s}}^{2}\right){\hat{\alpha }}_{2}; \end{array}$$ (20)

the edge conditions,

$$\frac{d}{d\bar{x}}\left(\bar{I}\frac{d^{2}{\hat{z}}_{f}}{d{\bar{x}}^{2}}\right)=0\;\mathrm{and}\;\bar{I}\frac{d^{2}{\hat{z}}_{f}}{d{\bar{x}}^{2}}=0\;\mathrm{at}\;\bar{x}={\bar{x}}_{t}=1,$$ (21)

$$d{\hat{z}}_{f}/d\bar{x}=0\;\mathrm{and}\;{\hat{z}}_{f}=0\;\mathrm{at}\;\bar{x}={\bar{x}}_{2}=0,$$ (22)

follow (6) and (7) by (11), (13) and (15).

Eq (20) has no closed-form analytical solution for ${\hat{z}}_{f}$, but it can be solved numerically, for example, by the Galerkin method [10]. To this end, ${\hat{z}}_{f}$ is written as

$${\hat{z}}_{f}\left(\bar{x}\right)=\sum_{m=1}^{\infty }{\hat{\zeta }}_{m}h_{m}\left(\bar{x}\right),$$ (23)

where *h*_1_, *h*_2_,… are basis functions on (0,1), each satisfying (21) and (22), whereas ${\hat{\zeta }}_{1}$, ${\hat{\zeta }}_{2}$,… are yet unknown coefficients. Because $\bar{I}\left(\bar{x}\right)$ vanishes faster than ${\left(1-\bar{x}\right)}^{2}$ as $\bar{x}\to 1$ (Appendix B), viable basis functions are

$$h_{n}\left(\bar{x}\right)={\bar{x}}^{n+1}.$$ (24)

If the sum in (23) is truncated, say, after *N* terms, ${\hat{z}}_{f}$ will probably not satisfy (20) at every point in the interval (0,1); nonetheless, the coefficients ${\hat{\zeta }}_{1},\ldots ,{\hat{\zeta }}_{N}$ can be chosen so as to make the residual orthogonal to the *N* basis functions retained in (23). Practically, it yields ${\hat{\zeta }}_{1},\ldots ,{\hat{\zeta }}_{N}$ as the solution of *N* algebraic equations,

$$\begin{array}{l} \sum_{n=1}^{N}\left({\left(i\bar{\omega }\right)}^{2}M_{mn}+i\bar{\omega }C_{mn}+K_{mn}^{\left(0\right)}+\bar{\kappa }K_{mn}^{\left(1\right)}\right){\hat{\zeta }}_{n}\\ \;=-{\hat{z}}_{2}\left(i\bar{\omega }A_{m}^{\left(0\right)}+{\left(i\bar{\omega }\right)}^{2}B_{m}^{\left(0\right)}\right)-{\hat{\alpha }}_{2}\left(A_{m}^{\left(0\right)}+i\bar{\omega }\left(A_{m}^{\left(1\right)}+2B_{m}^{\left(0\right)}\right)+{\left(i\bar{\omega }\right)}^{2}B_{m}^{\left(1\right)}\right) \end{array}$$ (25)

(*m* = 1,2,…,*N*), obtained by integrating (20), subject to (23), with *h*_1_,*h*_2_,…,*h*_*N*_ over (0,1). The coefficients *M*_*mn*_, *C*_*mn*_, $K_{mn}^{\left(0\right)}$, $K_{mn}^{\left(1\right)}$, $A_{m}^{\left(0\right)}$, $A_{m}^{\left(1\right)}$, $B_{m}^{\left(0\right)}$ and $B_{m}^{\left(1\right)}$ can be found in Appendix C.

Given ${\hat{\zeta }}_{1},\ldots ,{\hat{\zeta }}_{N}$, ${\hat{z}}_{t}$ and ${\hat{\alpha }}_{t}$ follow by (3), (4), (16–18) and (23):

$${\hat{z}}_{t}={\hat{z}}_{2}+{\hat{\alpha }}_{2}+\sum_{m=1}^{N}{\hat{\zeta }}_{m}h_{m}\left(1\right),$$ (26)

$${\hat{\alpha }}_{t}={\hat{\alpha }}_{2}+\sum_{m=1}^{N}{\hat{\zeta }}_{m}{\left(dh_{m}/d\bar{x}\right)}_{\bar{x}=1};$$ (27)

both expressions will be needed in the next section.

The combination of (5) and (9) (that leads to (10)) is comparable with the combination of Eqs (4) and (17) in Ref. [11]. Being linear, however, the present combination lends itself to a much simpler solution that will prove invaluable to the analysis of the following section.

## 4. Hydrodynamic Performance

### 4.1. Propulsion efficiency

The tail-beat-period-averaged thrust and power generated by the fish are

$$\langle T\rangle =\rho v^{2}s_{t}^{2}\langle \bar{T}\rangle ,$$ (28)

$$\langle P\rangle =\rho v^{3}s_{t}^{2}\langle \bar{P}\rangle ,$$ (29)

where the angular brackets mark a period-averaged quantity, whereas

$$\langle \bar{T}\rangle =\frac{\pi }{4}\left({\bar{\omega }}^{2}{\hat{z}}_{t}{\tilde{z}}_{t}-{\hat{\alpha }}_{t}{\tilde{\alpha }}_{t}\right),$$ (30)

〈P¯〉=π2(ω¯2z^tz˜t+ω¯Im(z˜tα^t)), (31)

are the respective reduced thrust and power, and the tilde marks a complex conjugate. The reader is referred to Appendix D (and to S1 File) for details. The key assumption underlying (30) and (31) is that either no wake is shed from the body anteriad of the caudal peduncle, or the vortices comprising that wake are weak. The propulsion efficiency follows (28–31) with

η=〈T〉v〈P〉=〈T¯〉〈P¯〉=12ω¯2z^tz˜t−α^tα˜tω¯2z^tz˜t+ω¯Im(z˜tα^t). (32)

The way $\langle \bar{T}\rangle$ and $\langle \bar{P}\rangle$ have been defined makes them explicitly independent of the geometry of the fin; they depend on it implicitly, through ${\hat{z}}_{t}$ and ${\hat{\alpha }}_{t}$ –see (26) and (27). Thus, given ${\hat{z}}_{2}$, ${\hat{\alpha }}_{2}$, $\bar{s}$, $\bar{I}$ and $\bar{\kappa }$, the combination of (30), (26) and (27) sets the reduced frequency needed to generate thrust $\langle \bar{T}\rangle$; in turn, given the frequency, the combination of (32), (26) and (27) sets the efficiency. Because the reduced stiffness changes with the swimming speed, the propulsion efficiency changes with both speed and thrust. Limiting cases where $\bar{\kappa }\to 0$ and $\bar{\kappa }\to \infty$ are addressed in Appendices E and F.

If *z*_*t*_ and *α*_*t*_ are in phase, Im(z˜tα^t) in the denominator of (32) vanishes, and the propulsion efficiency becomes $\left(1/2\right)\left(1-{\left|{\hat{\alpha }}_{t}/\bar{\omega }{\hat{z}}_{t}\right|}^{2}\right)$, less than one-half. To make a swimmer efficient, Im(z˜tα^t) should be negative. Essentially, this is the basis of the conjecture made at the beginning of the paper on the necessity of coordinated flex.

### 4.2. Tail-beat frequency

The drag of a swimmer can be expressed as

$$D=\frac{1}{2}\rho v^{2}SC_{D},$$ (33)

where *S* is an arbitrary reference area and *C*_*D*_ is the respective drag coefficient. It will prove convenient to choose *S* as the maximal cross-section area. In combination with (28), Eq (33) implies that in order to swim with constant speed, the reduced thrust should satisfy

$$\langle \bar{T}\rangle =\frac{S}{2s_{t}^{2}}C_{D};$$ (34)

the case of accelerated swimming is addressed in Appendix G. Because the drag coefficient is practically independent of speed (see S2 File), so is the reduced thrust needed to sustain it. Consequently, if ${\hat{z}}_{t}$ and ${\hat{\alpha }}_{t}$ were independent of speed, the reduced tail beat frequency would have been independent of speed as well (by (30)), implying proportionality between the ‘real’ tailbeat frequency and the swimming speed. Changes in the reduced stiffness make ${\hat{z}}_{t}$ and ${\hat{\alpha }}_{t}$, and hence the reduced frequency, speed dependent.

### 4.3. Swimming gaits

The swimming gait alters the way in which the propulsion efficiency is affected by flex of the caudal fin. The swimming gait is reflected in ${\hat{z}}_{2}$ and ${\hat{\alpha }}_{2}$, or, to be more specific, in the magnitude and argument of the ratio between the two. The manifestation of the gait in these two quantities can be elucidated by (temporary) assuming *z*_*b*_(*t*,*x*) = *ζ*(*x*)cos(*ωt* – 2*πx*/*λ*); it represents a backwards propagating wave of length *λ*, modulated by some function *ζ*. Under this assumption, ${\hat{z}}_{2}=\zeta \left(0\right)/l_{t}$ and ${\hat{\alpha }}_{2}={\left(d\zeta /dx-2\pi i\zeta /\lambda \right)}_{x=0}$ by (1), (13) and (16). Loosely following the classification of Breder [14], an idealized anguilliform gait can be associated with a short wave, (*dζ*/*dx*)_*x* = 0_ ≪ 2*π*(*ζ*/*λ*)_*x* = 0_; an idealized carangiform gait can associated with a long wave, (*dζ*/*dx*)_*x* = 0_ ≫ 2*π*(*ζ*/*λ*)_*x* = 0_. In the first case, $\arg\left({\hat{\alpha }}_{2}/{\hat{z}}_{2}\right)$ → −*π*/2; in the second case, $\arg\left({\hat{\alpha }}_{2}/{\hat{z}}_{2}\right)\to 0$. Realistic anguilliform, sub-carangiform and carangiform gaits span the range between these two extremes.

The recoil of the caudal fin at low sustained speeds is invariably small, because the forces acting on it are small. In this case, ${\hat{\alpha }}_{t}={\hat{\alpha }}_{2}$, ${\tilde{z}}_{t}={\tilde{z}}_{2}+{\tilde{\alpha }}_{2}$, and hence Im(α^tz˜t)=Im(α^2z˜2). If the swimmer were using a carangiform gait, for which $\arg\left({\hat{\alpha }}_{2}/{\hat{z}}_{2}\right)\to 0$, Im(α^tz˜t) would have vanished, and the efficiency (32) would have been less than one-half. If the swimmer were using an anguilliform gait, for which $\arg\left({\hat{\alpha }}_{2}/{\hat{z}}_{2}\right)$ → −*π*/2, Im(α^tz˜t) would have been negative, and its propulsion efficiency could have been as good as this gait allows. Fin’s recoil can potentially turn Im(α^tz˜t) negative, which is a big asset for a carangiform swimmer, but not necessarily an asset for an anguilliform one.

## 5. Results

### 5.1. Simulation parameters

As an example, consider a swimmer furnished with a flexible caudal fin, modelled after the soft-rayed fin of the blue tilapia *Oreochromis aureus* (Steindachner). It has a trapezoidal planform,

$$\bar{s}\left(\bar{x}\right)={\bar{s}}_{2}+\left(1-{\bar{s}}_{2}\right)\bar{x},$$ (35)

and its fractional area moment is

$$\bar{I}\left(\bar{x}\right)={\left(1-\bar{x}\right)}^{3};$$ (36)

the choice is justified in Appendix B.

**Fig 2:**
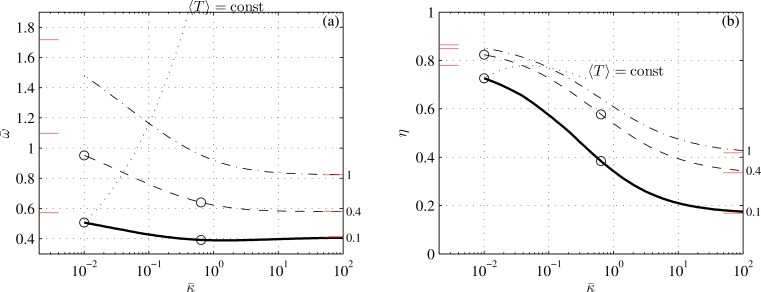
Reduced tailbeat frequency (a) and propulsion efficiency (b) of an idealized carangiform swimmer as functions of reduced stiffness at three reduced thrusts, indicated next to the respective lines. Short lines adjacent to the left and right margins mark asymptotic values from Appendices E and F. Circles mark the points shown in Fig 3. Conditions are those of case 1 in Table 1.

**Fig 3:**
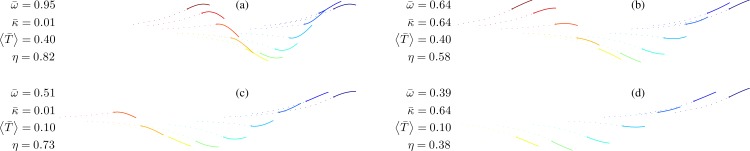
Simulated snapshots of the caudal fin during swimming. Direction of motion is from right to left. A dot marks the caudal peduncle. Conditions are those of case 1 in Table 1. Particular data is shown to the left of the respective figures. To emulate the fish motion, it was assumed that the length of the fish *l* equals 2*πl*_*t*_–under this assumption, the stride length, $l_{s}=2\pi l_{t}/\bar{\omega }=l/\bar{\omega }$, is one body length at $\bar{\omega }=1$.

**Fig 4:**
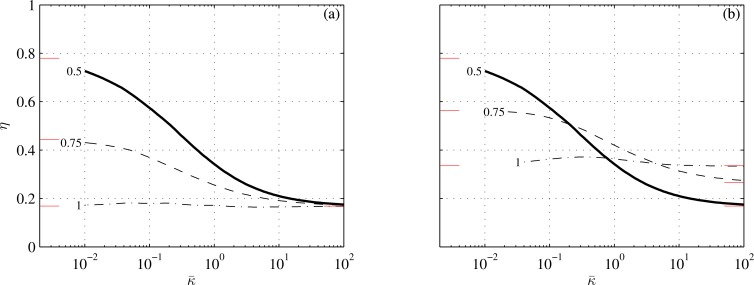
Propulsion efficiency as a function of reduced stiffness at three values of ${\bar{s}}_{2}$ (shown next to respective lines). Conditions are those of cases 2 and 3 in Table 1. In (a), *s*_*t*_ is constant; in (b), *s*_2_ is constant. The thick lines are the same as in Fig 2. Short lines adjacent to the left and right margins mark asymptotic values from Appendices E and F.

**Fig 5:**
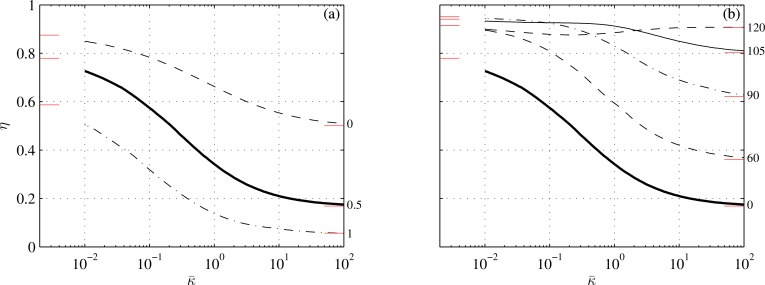
Propulsion efficiency as a function of reduced stiffness at three values of $\left|{\hat{\alpha }}_{2}/{\hat{z}}_{2}\right|$ at $\arg\left({\hat{\alpha }}_{2}/{\hat{z}}_{2}\right)=0$ (a) and five values of $\arg\left({\hat{\alpha }}_{2}/{\hat{z}}_{2}\right)$ at $\left|{\hat{\alpha }}_{2}/{\hat{z}}_{2}\right|=0.5$ (b). The thick line is the same as in Fig 2. Conditions are those of cases 4 and 5 in Table 1. Short lines adjacent to the left and right margins mark asymptotic values from Appendices E and F.

**Fig 6:**
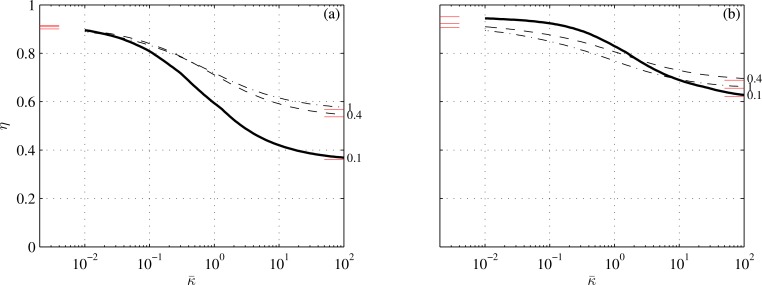
Propulsion efficiency as a function of reduced stiffness at three values of reduced thrust (indicated to the right of the respective lines). 60 degrees phase lag is on the left (a); 90 degrees is on the right (b). The thick lines on the two figures are the same as those marked ‘60’ and ‘90’ in Fig 5B. Conditions are those of cases 6 and 7 in Table 1. Short lines adjacent to the left and right margins mark asymptotic values from Appendices E and F.

With (35) and (36), all the matrices in (25) could have been found analytically; they are listed in Appendix C. The average thrust, power and propulsion efficiency have been computed with (26), (27), (30), (31) and (32) over a dense grid of tail-beat frequencies (201 values between 0.1 and 3) and reduced stiffnesses (101 values between 10^−2^ and 10^2^). They were recompiled *a posteriori* for three values of average reduced thrusts: 0.1, 0.4, and 1; the lowest of the three probably just offsets drag (Appendix G). Additional parameters are specified in Table 1. The results are shown in Figs 2–6. Fig 3 shows snapshots of four points from Fig 2; Fig 4 shows the effects of the caudal peduncle width; Figs 5 and 6 show the effects of the swimming gait. The effects of changing the exponent in (36) (to 2 or 4) and of replacing (35) with $\bar{s}\left(\bar{x}\right)={\left({\bar{s}}_{2}+\left(1-{\bar{s}}_{2}\right)\bar{x}\right)}^{1/n}$ (where *n* equals 2 or 3), turned unremarkable in any sense, and hence are not shown.

**Table 1:** Numerical cases addressed in the text.

case	${\bar{s}}_{2}$	$\left|{\hat{\alpha }}_{2}/{\hat{z}}_{2}\right|$	$-\arg\left({\hat{\alpha }}_{2}/{\hat{z}}_{2}\right)$	${\hat{z}}_{2}$	$\langle \bar{T}\rangle$
1	0.5	0.5	0	1.0	0.1,0.4,1
2	0.5,0.75,1	0.5	0	1.0	0.1
3	0.5,0.75,1	0.5	0	1.0	$0.1{\left({\bar{s}}_{2}/0.5\right)}^{2}$
4	0.5	0, 0.5, 1	0	1.0	0.1
5	0.5	0.5	0,*π*/3,*π*/2,7*π*/12,2*π*/3	1.0	0.1
6	0.5	0.5	*π*/3	1.0	0.1,0.4,1
7	0.5	0.5	*π*/2	1.0	0.1,0.4,1

### 5.2. Carangiform gait

The effects of caudal fin flexibility on hydrodynamic performance of an idealized carangiform swimmer (for which *z*_2_ and *α*_2_ are in phase) are shown in Figs 2–4. Perhaps the most conspicuous is Fig 2B that shows the effect of recoil on the propulsion efficiency at different fin loadings. At constant reduced thrust, the efficiency hardly changes when $\bar{\kappa }$ is below a few hundredths, or above a few units; it drops almost four-fold between these two regimes. Recall that $\bar{\kappa }\propto v^{-2}$ by (12). Hence, for a given fin geometry, small and large values of $\bar{\kappa }$ correspond to high and low swimming speeds respectively. Because the swimming speed of a fish can change from less than 1 body length a second at cruise to roughly 10 body lengths a second at burst [15], the caudal fin that is designed to reach the highest possible speed at maximum power, is necessarily inefficient at low speed cruise. Increasing the thrust at low speed to the same thrust that would have yielded $\bar{\kappa }=0.01$ and $\langle \bar{T}\rangle =0.1$ at the terminal speed, practically restores the propulsion efficiency–see the line marked ‘〈*T*〉 = const’. Thrust-dependent efficiency of a flexible fin may explain the advantages of burst-and-coast locomotion strategies, where high thrust–and hence more efficient–bursts are alternated with unpowered glides [9].

The four plates of Fig 3 show simulated fin shapes at four swimming conditions marked by circles on Fig 2; the plates are arranged in the same order as the circles. Fig 3C reflects what can be considered a high-speed burst. The reduced thrust is 0.1, which is probably just enough to offset drag; the efficiency is 0.73. In Fig 3D, the reduced thrust is the same, but the stiffness of the fin has been increased 64-fold, reflecting an equivalent 8-fold decrease in the swimming speed. Because the thrust (‘real’ thrust, not the reduced one) is lower than in the previous case, the fin flexes less and is less efficient. In Fig 3A and 3B, the respective reduced stiffnesses are same as in Fig 3C and 3D, but the tailbeat frequency has been increased roughly two-fold to obtain four times the respective thrusts; in both cases, the fish would have been accelerating. The fin flexes more, and is more efficient.

The propulsion efficiency of a carangiform swimmer with an infinitely rigid caudal fin ($\bar{\kappa }\to \infty$) drops below 1/2 and becomes independent of the shape of the fin (Appendix F). One may rightly ask why the propulsion efficiency of a carangiform swimmer with an infinitely soft fin ($\bar{\kappa }\to 0$), which can withstand no force, is better than the efficiency of the same swimmer with no caudal fin at all? Under present set of assumptions, the latter should have been the same as in the case $\bar{\kappa }\to \infty$. The answer lays in the leading edge suction–the force acting on the dorsal and ventral edges of the caudal fin–and in limitations of the present theory. Vanishing of the lateral loading does not imply vanishing of the leading edge suction. Since the latter was not a part of hydrodynamic loads flexing the fin–it was deemed a higher order effect when constructing Eq (5)–thrust could be obtained without collapsing the fin. The leading edge suction disappears when *s*_2_ = *s*_*t*_ (Fig 4A), and indeed the propulsion efficiency of a carangiform swimmer furnished with a soft fin becomes the same as the efficiency of this swimmer with no caudal fin at all. Cases with $\bar{\kappa }$ smaller than, say, 0.01, are inconsistent with the assumptions underlying them (Appendix A) and hence are not shown in any of the figures.

Propulsion efficiency significantly increases with decreasing body angle at the caudal peduncle (Fig 5A). An explanation can be based on (32). At small and moderate values of $\bar{\kappa }$ –when the fin flexes appreciably–the efficiency depends mainly on the sign and magnitude of Im(z˜tα^t) in the denominator. To make a fin efficient Im(z˜tα^t) should be negative and large. To make Im(z˜tα^t) negative *α*_*t*_ should lag 90 degrees behind, and to this end the fin should flex. Because in an idealized carangiform gait, *α*_2_ and *z*_2_ are in phase, the smaller ${\hat{\alpha }}_{2}$ is, the more negative Im(z˜tα^t) can become for the same flex.

### 5.3. Anguilliform and subcarangiform gaits

Im(z˜tα^t) can be made more negative without flexing the fin at all by lagging *α*_2_ behind *z*_2_ through timely actuation of the tail muscles. Indeed, the propulsion efficiency dramatically increases with the phase angle between *z*_2_ and *α*_2_ (Fig 5B). 90 degrees lag yields the best efficiency with a soft fin; 120 degrees yields the best efficiency with a stiff fin. Larger lag needed with a stiff fin is formally justified in Appendix F, but can be accepted plausible because it is *α*_*t*_ that should lag 90 degrees behind *z*_*t*_, rather than *α*_2_ behind *z*_2_.

The propulsion efficiency of an idealized anguilliform gait, where *α*_2_ lags 90 degrees behind *z*_2_, is insensitive to the rigidity of the fin, and, concurrently, to thrust (Fig 6B). Consequently, burst-and-coast strategies offer no advantage to anguilliform swimmers, and to the best of our knowledge, no swimmer of this gait has been observed using them. Reducing the phase angle between *α*_2_ and *z*_2_ restores the dependence of the propulsion efficiency on thrust (Fig 6A), and hence some subcarangiform swimmers may benefit from burst-and-coast strategies.

## 6. Discussion

Anguilliform swimmers do not need a flexible caudal fin to be hydrodynamically efficient, and elastic recoil of the fin has practically no effect on their propulsion efficiency. Carangiform swimmers do need it. The hypothesis that the flex of the caudal fin is passive infers dependence of their propulsion efficiency on the fin loading. When thrust offsets drag–as happens when swimming at constant speed–the propulsion efficiency can change almost four-fold over a ten-fold change of speed. Accordingly, a passively flexing fin that is optimized to provide a carangiform swimmer with the best efficiency at high speeds will necessarily be inefficient at low speeds, where the swimmer spends most of its time.

In principle, the loss of propulsion efficiency at low speeds can be compensated by swimming in short powerful (and hence efficient) bursts, alternated by effortless glides; nonetheless, carangiform swimmers have been observed sustaining low swimming speeds [16,17]. It implies one of the following: (i) they swim inefficiently at low speeds; (ii) their caudal fin is optimized to provide the best efficiency at low speeds–and hence is inefficient at high speeds; or (iii) the flex of the fin is not passive.

The first two alternatives are possible, but hardly probable. We must therefore conclude that carangiform and, possibly, some subcarangiform swimmers do have at least some control over the flex of the caudal fin. Referring to [4], actinopterygian fish have a complex set of (slow, aerobic) tail muscles, some connected to the distal raylets of the caudal fin, and some connected to its left and right hemitrichia. The former can spread or fold the fin; the latter can actively flex it [5,18].

Decreasing the span of the fin (2*s*_*t*_) for given thrust and area moment increases both the reduced thrust $\langle \bar{T}\rangle$ and the reduced stiffness $\bar{\kappa }$ –see (28) and (12). The combined effect is shown in Fig 4B. Folding the fin reduces the efficiency at high speeds, where $\bar{\kappa }$ is small, but improves it at low speeds, where $\bar{\kappa }$ is large. The loss of efficiency at high speeds has can be justified by the loss of the leading edge suction on the anterior margins of the fin; the gain at low speeds can be justified by the increase in the reduced thrust–see Eq (F8) in Appendix F. In any case, the magnitude of this gain, albeit significant, cannot make a carangiform swimmer that is optimized for high speeds, efficient at low speeds.

There is no doubt that the muscles that are directly attached to the hemitrichia can increase the flex at low speeds by pulling against the structure of the fin, together with the hydrodynamic forces. Significant activity of the tail muscles in slow swimming tilapia [4,5], occurring ipsilateral with the displacement of the caudal peduncle during the beginning of a stroke seems to support this conjecture. With proper flex, the propulsion efficiency can be fully restored and kept independent of the swimming conditions. Nonetheless, being small and slow, the tail muscles may lack the speed and power to flex the fin at high speeds. Whenever the proper flex of the fin cannot be achieved, the propulsion efficiency will return to increase with thrust; whenever the propulsion efficiency increases with thrust, burst-and-coast strategies become energetically advantageous [9].

## Appendix A–Applicability Limits

Eq (5) manifests a formal leading (linear) order approximation with respect to *z*_*b*_. The contribution

$$\Delta \left(t,x\right)=-\frac{\partial^{2}z_{b}\left(t,x\right)}{\partial x^{2}}\int_{x}^{x_{t}}f_{∥}\left(t,x^{\prime }\right)dx^{\prime }=-\frac{\partial^{2}z_{f}\left(t,x\right)}{\partial x^{2}}\int_{x}^{x_{t}}f_{∥}\left(t,x^{\prime }\right)dx^{\prime }$$ (A1)

of the in-plane component of the hydrodynamic force,

$$f_{∥}\left(t,x\right)=-\rho \frac{\pi }{2}{\left(\frac{Dz_{b}\left(t,x\right)}{Dt}\right)}^{2}\frac{ds^{2}\left(x\right)}{dx},$$ (A2)

that has been tacitly omitted in (5), is formally a second order term with respect to *z*_*b*_. Eq (A2) can be found in S1 File–see, in particular, Eq (S8) thereat.

To keep (5) coherent, Δ(*t*,*x*) should remain small relative to the only (linear with respect to *z*_*b*_) term eventually retained on its right hand side, *f*_⊥_(*t*,*x*). In turn, if (5) is coherent, then

$$f_{⊥}\left(t,x\right)=\frac{\partial^{2}}{\partial x^{2}}\left(EI\left(x\right)\frac{\partial^{2}z_{f}\left(t,x\right)}{\partial x^{2}}\right).$$ (A3)

To have |Δ(*t*,*x*)| ≪ |*f*_⊥_(*t*,*x*)| one needs

$$\rho \frac{\pi }{2}\left|\frac{\partial^{2}z_{f}\left(t,x\right)}{\partial x^{2}}\int_{x}^{x_{t}}{\left(\frac{Dz_{b}\left(t,x^{\prime }\right)}{Dt}\right)}^{2}\frac{ds^{2}\left(x^{\prime }\right)}{dx^{\prime }}dx^{\prime }\right|\ll \left|\frac{\partial^{2}}{\partial x^{2}}\left(EI\left(x\right)\frac{\partial^{2}z_{f}\left(t,x\right)}{\partial x^{2}}\right)\right|.$$ (A4)

Using (11–15) it can be brought into the dimensionless form

$$\left|\frac{\partial^{2}{\bar{z}}_{f}\left(\bar{t},\bar{x}\right)}{\partial {\bar{x}}^{2}}\int_{\bar{x}}^{1}{\left(\frac{\partial {\bar{z}}_{b}\left(\bar{t},{\bar{x}}^{\prime }\right)}{\partial \bar{t}}+\frac{\partial {\bar{z}}_{b}\left(\bar{t},{\bar{x}}^{\prime }\right)}{\partial {\bar{x}}^{\prime }}\right)}^{2}\frac{d{\bar{s}}^{2}\left(\bar{x^{\prime }}\right)}{d\bar{x^{\prime }}}d\bar{x^{\prime }}\right|\ll 2\bar{\kappa }\left|\frac{\partial^{2}}{\partial {\bar{x}}^{2}}\left(\bar{I}\left(\bar{x}\right)\frac{\partial^{2}{\bar{z}}_{f}\left(\bar{t},\bar{x}\right)}{\partial {\bar{x}}^{2}}\right)\right|.$$ (A5)

The expression on the left is of the order of ${\left(\frac{\partial {\bar{z}}_{b}}{\partial \bar{t}}+\frac{\partial {\bar{z}}_{b}}{\partial {\bar{x}}^{\prime }}\right)}^{2}\left|{\bar{z}}_{f}\right|$; the expression on the right is of the order of $\bar{\kappa }\left|{\bar{z}}_{f}\right|$. In other words, Eq (5) is coherent if ${\left(\frac{\partial {\bar{z}}_{b}}{\partial \bar{t}}+{\bar{\alpha }}_{b}\right)}^{2}\ll \bar{\kappa }$.

## Appendix B–Caudal Fin of the Blue Tilapia

Caudal skeleton of the Nile tilapia *Oreochromis niloticus* (Linnaeus) was described in exhaustive details in Ref. [18]. Particular measurements reported below were made using a caliper on a specimen of the blue tilapia *Oreochromis aureus* (Steindachner), purchased at the local market. The outline of the fin was practically trapezoidal, with *s*_2_ ≈ 17.5 mm, *s*_*t*_ ≈ 35 mm, and *x*_*t*_–*x*_2_ ≈ 70 mm. The thickness *θ* and the width *w* of each hemitrichion changed almost linearly along the ray; *θ* was 0.8 mm at the proximal end and 0.02 mm at the distal end, *w* was 0.8 mm at the proximal end and 4 mm at the distal end. The proximal parts of the hemitrichia were 1 mm apart, separated by a soft tissue; the distal parts were fused. For large part of the fin, the distance *h* between the hemitrichia changed linearly along the fin. Formally, these observations imply

$$h\left(\bar{x}\right)\propto 1-\bar{x},\;\theta \left(\bar{x}\right)\propto 1-\bar{x},\;w\left(\bar{x}\right)\propto \bar{x}+b,$$ (B1)

where $\bar{x}\in \left(0,1\right)$ is the reduced coordinate along the fin and *b* > 0 is a certain constant.

The area moment of the fin can be roughly approximated as the area moment of a single ray times the number of rays. In turn, because the soft tissue separating the hemitrichia allows them to move one relative to the other, the area moment of a single ray should be less than the area moment of the two hemitrichia held at a fixed distance one from the other, but more than twice the area moment of one hemitrichion. The former is proportional to *wθh*^2^, the latter is proportional to *wθ*^3^; in view of (B1) both behave as $\left(\bar{x}+b\right){\left(1-\bar{x}\right)}^{3}$, suggesting that the area moment of the fin, $\bar{I}\left(\bar{x}\right)$, should behave the same. Guided by computational convenience, the behavior of $\bar{I}\left(\bar{x}\right)$ was approximated by ${\left(1-\bar{x}\right)}^{p}$, where *p* ∈ (2,4).

## Appendix C–Coefficients in (25)

The coefficients in (25) are:

$$K_{mn}^{\left(0\right)}=\int_{0}^{1}h_{m}\frac{d}{d\bar{x}}\left({\bar{s}}^{2}\frac{dh_{n}}{d\bar{x}}\right)d\bar{x}={\left(h_{m}\frac{dh_{n}}{d\bar{x}}\right)}_{\bar{x}=1}-\int_{0}^{1}{\bar{s}}^{2}\frac{dh_{m}}{d\bar{x}}\frac{dh_{n}}{d\bar{x}}d\bar{x},$$ (C1)

$$K_{mn}^{\left(1\right)}=\int_{0}^{1}h_{m}\frac{d^{2}}{d{\bar{x}}^{2}}\left(\bar{I}\frac{d^{2}h_{n}}{d{\bar{x}}^{2}}\right)d\bar{x}=\int_{0}^{1}\bar{I}\frac{d^{2}h_{m}}{d{\bar{x}}^{2}}\frac{d^{2}h_{n}}{d{\bar{x}}^{2}}d\bar{x},$$ (C2)

$$C_{mn}=\int_{0}^{1}h_{m}\left(\frac{d}{d\bar{x}}\left(h_{n}{\bar{s}}^{2}\right)+{\bar{s}}^{2}\frac{dh_{n}}{d\bar{x}}\right)d\bar{x}={\left(h_{m}h_{n}\right)}_{x=1}+\int_{0}^{1}{\bar{s}}^{2}\left(h_{m}\frac{dh_{n}}{d\bar{x}}-h_{n}\frac{dh_{m}}{d\bar{x}}\right)d\bar{x},$$ (C3)

$$M_{mn}=\int_{0}^{1}{\bar{s}}^{2}h_{n}h_{m}d\bar{x},$$ (C4)

$$A_{m}^{\left(k\right)}=\int_{0}^{1}\frac{d{\bar{s}}^{2}}{d\bar{x}}h_{m}{\bar{x}}^{k}d\bar{x},$$ (C5)

$$B_{m}^{\left(k\right)}=\int_{0}^{1}{\bar{s}}^{2}h_{m}{\bar{x}}^{k}d\bar{x}.$$ (C6)

(C1–C3) follow (20) by a few integrations by parts, which exploit the assumption that *h*_1_, *h*_2_, … satisfy (21) and (22) identically, and that $\bar{s}\left(1\right)=1$ by (14).

When $h_{n}\left(\bar{x}\right)={\bar{x}}^{n+1}$, $\bar{s}\left(x\right)={\bar{s}}_{2}+\left(1-{\bar{s}}_{2}\right)\bar{x}$ and $\bar{I}\left(\bar{x}\right)={\left(1-\bar{x}\right)}^{p}$, and in which *n* and *p* are positive integers, the integrals in (C1–C6) can be evaluated analytically. The result is

$$K_{mn}^{\left(0\right)}=n+1-\left(m+1\right)\left(n+1\right)\left(\frac{{\bar{s}}_{2}^{2}}{m+n+1}+\frac{2{\bar{s}}_{2}\left(1-{\bar{s}}_{2}\right)}{m+n+2}+\frac{{\left(1-{\bar{s}}_{2}\right)}^{2}}{m+n+3}\right),$$ (C7)

$$K_{mn}^{\left(1\right)}=\left(m+1\right)m\left(n+1\right)n\sum_{k=0}^{p}\left(\begin{array}{l} p\\ k \end{array}\right)\frac{{\left(-1\right)}^{k}}{m+n+k-1},$$ (C8)

$$C_{mn}=1+\left(n-m\right)\left(\frac{{\bar{s}}_{2}^{2}}{n+m+2}+\frac{2{\bar{s}}_{2}\left(1-{\bar{s}}_{2}\right)}{n+m+3}+\frac{{\left(1-{\bar{s}}_{2}\right)}^{2}}{n+m+4}\right),$$ (C9)

$$M_{mn}=\frac{{\bar{s}}_{2}^{2}}{n+m+3}+\frac{2{\bar{s}}_{2}\left(1-{\bar{s}}_{2}\right)}{n+m+4}+\frac{{\left(1-{\bar{s}}_{2}\right)}^{2}}{n+m+5},$$ (C10)

$$A_{m}^{\left(k\right)}=\frac{2{\bar{s}}_{2}\left(1-{\bar{s}}_{2}\right)}{k+m+2}+\frac{2{\left(1-{\bar{s}}_{2}\right)}^{2}}{k+m+3},$$ (C11)

$$B_{m}^{\left(k\right)}=\frac{{\bar{s}}_{2}^{2}}{k+m+2}+\frac{2{\bar{s}}_{2}\left(1-{\bar{s}}_{2}\right)}{k+m+3}+\frac{{\left(1-{\bar{s}}_{2}\right)}^{2}}{k+m+4}.$$ (C12)

## Appendix D–Power, Thrust and Efficiency

Adopting the elongated body theory, and assuming that no wakes are released anteriad of the caudal fin, the period-averaged power and thrust are given

$$\langle P\rangle =\rho v\pi s_{t}^{2}{\left\langle \frac{\partial z_{b}}{\partial t}\frac{Dz_{b}}{Dt}\right\rangle }_{x=x_{t}},$$ (D1)

$$\langle T\rangle =\rho \frac{\pi }{2}s_{t}^{2}{\left\langle {\left(\frac{\partial z_{b}}{\partial t}\right)}^{2}-v^{2}{\left(\frac{\partial z_{b}}{\partial x}\right)}^{2}\right\rangle }_{x=x_{t}},$$ (D2)

where *D*/*Dt* = ∂/∂*t* + *v*∂/∂*x* is the convective derivative. Essentially, these are Eqs (26) and (25) in Ref. [12]–under the present assumptions, the respective second and third terms in these equations cancel out. (D1) and (D2) are formally derived in S1 File–see Eqs (S14) and (S20) thereat.

The particular case that will be needed for this study is the case where the motion of the swimmer, manifested in *z*_*b*_, is harmonic. Consistent with (11), (13), and (16–19) we set

zb(t,x)=zb(t¯lt/v,x¯lt)=ltRe(z^b(x¯)eiω¯t¯), (D3)

αb(t,x)=∂zb(t,x)∂x=Re(α^b(x¯)eiω¯t¯). (D4)

With these,

⟨∂zb∂tDzbDt⟩=12v2ω¯(ω¯z^bz˜b+Im(z˜bα^b)), (D5)

$$\left\langle {\left(\frac{\partial z_{b}}{\partial t}\right)}^{2}-v^{2}{\left(\frac{\partial z_{b}}{\partial x}\right)}^{2}\right\rangle =\frac{1}{2}v^{2}\left({\bar{\omega }}^{2}{\hat{z}}_{b}{\tilde{z}}_{b}-{\hat{\alpha }}_{b}{\tilde{\alpha }}_{b}\right);$$ (D6)

the tilde marking a complex conjugate. Consequently,

⟨P⟩=ρv3π2st2(ω¯2z^tz˜t+ω¯Im(z˜tα^t)), (D7)

$$\left\langle T\right\rangle =\rho v^{2}\frac{\pi }{4}s_{t}^{2}\left({\bar{\omega }}^{2}{\hat{z}}_{t}{\tilde{z}}_{t}-{\hat{\alpha }}_{t}{\tilde{\alpha }}_{t}\right),$$ (D8)

by (D1) and (D2). In these, ${\hat{z}}_{t}={\hat{z}}_{b}\left({\bar{x}}_{t}\right)$ and ${\hat{\alpha }}_{t}={\hat{\alpha }}_{b}\left({\bar{x}}_{t}\right)$.

The propulsion efficiency will be defined as the ratio of the power made good, 〈*T*〉*v*, and the power spent, 〈*P*〉; that is,

η=⟨T⟩v/⟨P⟩. (D9)

Its explicit form,

η=12ω¯2z^tz˜t−α^tα˜tω¯2z^tz˜t+ω¯Im(z˜tα^t), (D10)

follows by (D7) and (D8).

## Appendix E–Case $\bar{\kappa }\to 0$

When $\bar{\kappa }\to 0$, the left-hand side of (5) vanishes identically, leaving *z*_*b*_ to be determined by

$${\bar{f}}_{⊥}\left(t,x\right)=0,$$ (E1)

subject to the edge conditions,

$$z_{b}\left(t,0\right)=z_{2}\left(t\right),$$ (E2)

$${\left(\frac{\partial z_{b}\left(t,x\right)}{\partial x}\right)}_{x=0}=\alpha_{2}\left(t\right).$$ (E3)

In (E1),

$${\bar{f}}_{⊥}\left(t,x\right)=-\frac{\pi }{s_{t}^{2}v^{2}}\frac{D}{Dt}\left(s^{2}\left(x\right)\frac{Dz_{b}\left(t,x\right)}{Dt}\right)$$ (E4)

by (8). As shown in Appendix A, this case is incoherent with the assumptions underlying (5), but it serves to verify the numerical solution.

Introducing (11–14), and assuming

z¯b(t¯,x¯)=Re(z^b(x¯)eiω¯t¯), (E5)

(E4) becomes

f¯⊥(t,x)=−πRe(eiω¯t¯(iω¯+ddx¯)(s¯2(x¯)(iω¯z^b(x¯)+dz^b(x¯)dx¯))). (E6)

It can be verified by direct substitution that (E6) is equivalent to

f¯⊥(t,x)=−πRe(eiω¯t¯−iω¯x¯ddx¯(s¯2(x¯)ddx¯(eiω¯x¯z^b(x¯)))). (E7)

Consequently, (E1) will be satisfied if

$$\frac{d}{d\bar{x}}\left(e^{i\bar{\omega }\bar{x}}{\hat{z}}_{b}\left(\bar{x}\right)\right)=\frac{\hat{a}}{{\bar{s}}^{2}\left(\bar{x}\right)},$$ (E8)

where $\hat{a}$ is a constant to be determined. Integrating (E8) on $\left(0,\bar{x}\right)$, one will find that

$${\hat{z}}_{b}\left(\bar{x}\right)=e^{-i\bar{\omega }\bar{x}}\left({\hat{z}}_{2}+\hat{a}\int_{0}^{\bar{x}}\frac{d\bar{x^{\prime }}}{{\bar{s}}^{2}\left(\bar{x^{\prime }}\right)}\right)$$ (E9)

satisfies both (E8) and the variant ${\hat{z}}_{b}\left(0\right)={\hat{z}}_{2}$ of (E2). Its derivative

$${\hat{\alpha }}_{b}\left(\bar{x}\right)=\frac{d{\hat{z}}_{b}\left(\bar{x}\right)}{d\bar{x}}=\frac{\hat{a}e^{-i\bar{\omega }\bar{x}}}{{\bar{s}}^{2}\left(\bar{x}\right)}-i\omega {\hat{z}}_{b}\left(\bar{x}\right)$$ (E10)

can be used in conjunction with (E3) to find $\hat{a}$. The variant of (E3) to this end is ${\hat{\alpha }}_{b}\left(0\right)={\hat{\alpha }}_{2}$; see (E5) and (16). Hence,

$$\hat{a}={\bar{s}}_{2}^{2}\left({\hat{\alpha }}_{2}+i\bar{\omega }{\hat{z}}_{2}\right)$$ (E11)

by (E10), and, consequently,

$${\hat{\alpha }}_{b}\left(\bar{x}\right)=\frac{{\bar{s}}_{2}^{2}e^{-i\bar{\omega }\bar{x}}}{{\bar{s}}^{2}\left(\bar{x}\right)}\left({\hat{\alpha }}_{2}+i\bar{\omega }{\hat{z}}_{2}\right)-i\omega {\hat{z}}_{b}\left(\bar{x}\right).$$ (E12)

Substituting (E12) and (E9) in (D7) yields

⟨P¯⟩=π2ω¯Im(z˜2a^)=π2s¯22(ω¯Im(z˜2α^2)+ω2|z^2|2), (E13)

as if the fish had no caudal fin; substituting them in (D8) yields

⟨T¯⟩=π4(ω¯2z^tz˜t−α^tα˜t)=−π4(a˜a^+iω¯(z˜2a^−a˜z^2))=π4s¯22((2−s¯22)ω¯2|z^2|2−s¯22|α^2|2+2(1−s¯22)ω¯Im(α^2z˜2)), (E14)

which exceeds the thrust that could have been obtained with no caudal fin as long as ${\bar{s}}_{2}<1$; the two are equal (and hence the efficiencies are equal) only when ${\bar{s}}_{2}=1$. The difference is attributed to the leading edge suction on the fin’s dorsal and ventral edges, neglected in (5). The underlying notion is that thrust, power, and efficiency of a fish with no caudal fin are given by the respective variants of (30), (31) and (32) with ${\hat{z}}_{2}$ and ${\hat{\alpha }}_{2}$ replacing ${\hat{z}}_{t}$ and ${\hat{\alpha }}_{t}$.

The tail-beat frequency needed to generate thrust $\left\langle \bar{T}\right\rangle$ is

ω¯=−1−s¯222−s¯22Im(α^2z˜2)|z^2|2+((1−s¯222−s¯22Im(α^2z˜2)|z^2|2)2+4⟨T¯⟩+πs¯24|α^2|2πs¯22(2−s¯22)|z^2|2)1/2 (E15)

by (E14); the power needed to this end is:

⟨P¯⟩=22−s¯22(⟨T¯⟩+π4s¯24|α^2|2+π4s¯24ω¯Im(α^2z˜2)) (E16)

by (E13) and (E14). To avoid obtaining an unwieldy expression, $\bar{\omega }$, which is given by (E15), have been left unassigned in (E16).

## Appendix F–Case *κ*→∞

In this case, ${\hat{\alpha }}_{t}={\hat{\alpha }}_{2}$, and, consequently, ${\hat{z}}_{t}={\hat{z}}_{2}+{\hat{\alpha }}_{2}$. The power, thrust and efficiency are

⟨P¯⟩=π2(ω¯2|z^2+α^2|2+ω¯Im(z˜2α^2)), (F1)

$$\left\langle \bar{T}\right\rangle =\frac{\pi }{4}\left({\bar{\omega }}^{2}{\left|{\hat{z}}_{2}+{\hat{\alpha }}_{2}\right|}^{2}-{\left|{\hat{\alpha }}_{2}\right|}^{2}\right),$$ (F2)

η=⟨T¯⟩⟨P¯⟩=12(1−|α^2|2+ω¯Im(z˜2α^2)ω¯2|z^2+α^2|2+ω¯Im(z˜2α^2)) (F3)

by (30–32). Eq (F2) can be inverted to obtain the tailbeat frequency:

$${\bar{\omega }}^{2}=\frac{4\left\langle \bar{T}\right\rangle }{\pi {\left|{\hat{z}}_{2}+{\hat{\alpha }}_{2}\right|}^{2}}+\frac{{\left|{\hat{\alpha }}_{2}\right|}^{2}}{{\left|{\hat{z}}_{2}+{\hat{\alpha }}_{2}\right|}^{2}};$$ (F4)

in turn, introducing (F4) in (F3) yields the propulsion efficiency:

η=12(1+π4|α^2|2⟨T¯⟩+Im(z˜2α^2)|z^2+α^2|⟨T¯⟩(π4(⟨T¯⟩+π4|α^2|2))1/2)−1. (F5)

It can be somewhat simplified by replacing ${\hat{z}}_{2}$ and ${\hat{\alpha }}_{2}$ with

$${\hat{z}}_{2}=\left|{\hat{z}}_{2}\right|e^{i\phi_{z}},\;{\hat{\alpha }}_{2}=\left|{\hat{\alpha }}_{2}\right|e^{i\left(\phi_{z}-\phi \right)},$$ (F6)

where *ϕ*_*z*_ is an arbitrary phase and *ϕ* is the phase angle between ${\hat{\alpha }}_{2}$ and ${\hat{z}}_{2}$; the result is

$$\eta =\frac{1}{2}{\left(1+\frac{\pi }{4}\frac{{\left|{\hat{\alpha }}_{2}\right|}^{2}}{\left\langle \bar{T}\right\rangle }-\frac{\left|{\hat{\alpha }}_{2}\right|\sin\phi }{\left\langle \bar{T}\right\rangle }{\left(\frac{{\left|{\hat{z}}_{2}\right|}^{2}\frac{\pi }{4}\left(\left\langle \bar{T}\right\rangle +\frac{\pi }{4}{\left|{\hat{\alpha }}_{2}\right|}^{2}\right)}{{\left|{\hat{z}}_{2}\right|}^{2}+{\left|{\hat{\alpha }}_{2}\right|}^{2}+2\left|{\hat{z}}_{2}\right|\left|{\hat{\alpha }}_{2}\right|\cos\phi }\right)}^{1/2}\right)}^{-1}.$$ (F7)

In an idealized carangiform swimming gait, *ϕ* = 0, in which case (F7) reduces to

$$\eta =\frac{1}{2}{\left(1+\frac{\pi }{4}\frac{{\hat{\alpha }}_{2}^{2}}{\left\langle \bar{T}\right\rangle }\right)}^{-1}.$$ (F8)

It is less than 0.5, decreasing with the angle of the body at the caudal peduncle and increasing with thrust.

The phase angle that maximizes the efficiency can be found by differentiating (F7) with respect to *ϕ* and equating the result to zero. It yields

$$\phi =\cos^{-1}\left(-\left|{\hat{\alpha }}_{2}\right|/\left|{\hat{z}}_{2}\right|\right),$$ (F9)

in excess of 90 degrees. The respective efficiency is

$$\underset{\phi }{\max}\eta =\frac{1}{2}{\left(1+\frac{\pi }{4}\frac{{\left|{\hat{\alpha }}_{2}\right|}^{2}}{\left\langle \bar{T}\right\rangle }-{\left(\frac{\pi }{4}\frac{{\left|{\hat{\alpha }}_{2}\right|}^{2}}{\left\langle \bar{T}\right\rangle }\left(1+\frac{\pi }{4}\frac{{\left|{\hat{\alpha }}_{2}\right|}^{2}}{\left\langle \bar{T}\right\rangle }\right)\right)}^{1/2}\right)}^{-1}$$ (F10)

by (F7).

## Appendix G–Excess Thrust

The reduced thrust needed to generate acceleration *a* is

$$\left\langle \bar{T}\right\rangle =\frac{D}{\rho v^{2}s_{t}^{2}}+\frac{m_{a}a}{\rho v^{2}s_{t}^{2}};$$ (G1)

it follows the second Newton law by (28). Here, *D* is the hydrodynamic drag and *m*_*a*_ is the apparent mass of the fish in the swimming direction. The apparent mass of a neutrally buoyant fish can be expressed as

$$m_{a}=k_{a}k_{m}\rho Sl,$$ (G2)

where *k*_*a*_ is the ratio between the apparent and real masses of the fish, *S* is its maximal cross section area, *l* is the body length, and *k*_*m*_ is the prismatic coefficient, the ratio between the volume occupied by the body and the minimal cylinder enclosing it. For a streamlined fish, *k*_*a*_ is practically unity; for most fishes, *k*_*m*_ is not significantly different from 0.5 [19].

The drag of the fish is commonly expressed by (33). We rewrite it here as

$$D=\frac{1}{2}\rho v^{2}SC_{D},$$ (G3)

where *S* is set the same as in (G2). With this choice of the reference area, *C*_*D*_ should be between 0.1 and 0.2 for most fishes (see S2 File); the exact value is inconsequential to the course of the discussion. Substituting (G2), (G3) and (30) in (G1) yields

$$\left\langle \bar{T}\right\rangle =\frac{S}{2s_{t}^{2}}\left(C_{D}+2k_{m}\frac{la}{v^{2}}\right).$$ (G4)

Because $S/2s_{t}^{2}$ is of the order of unity, $\left\langle \bar{T}\right\rangle$ of the order of 0.1 will keep a fish swimming at constant speed; $\left\langle \bar{T}\right\rangle$ of the order of unity should be enough to accelerate it.

## Supporting Information

S1 File Recapitulation of the slender body theory. (PDF)

S2 File Drag coefficient of a fish. (PDF)
