# Supplementary material for: Hydrodynamics of a Flexible Soft-Rayed Caudal Fin
Source: PLoS One. 2016 Oct 3;11(10):e0163517. doi: 10.1371/journal.pone.0163517 (PMC5047476; doi:10.1371/journal.pone.0163517)
Supplement: S1 File — (PDF) [file pone.0163517.s001.pdf]

Slender body theory

This document summarizes the basic formulae of the slender body theory. It is based mainly on Ref [1].

Consider a slender fish, swimming with constant velocity v along a straight path. ρ is the density of water, l is an arbitrary reference length associated with the fish (in the manuscript it was chosen as the length of the caudal fin). Unless explicitly indicated otherwise, all other quantities defined below will be dimensionless, based on l, v, l/v, v/l, $\rho v^2 l$, $\rho v^2 l^2$, $\rho v^3 l$ and $\rho v^3 l^2$ as the respective units of length, velocity, time, frequency, force per unit length, force, power per unit length and power, respectively.

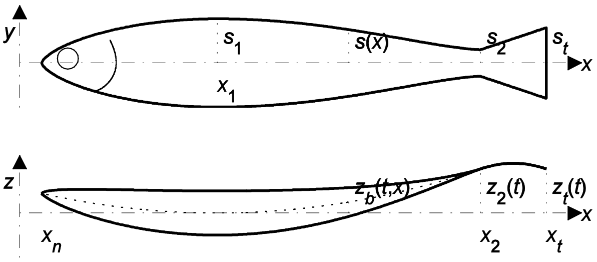

Fig S1. The reference frame and the notation.

A right-handed reference frame will follow the fish as it swims, the x- and y-axes pointing backwards, along the swimming path, and upwards, parallel to the flat side of the caudal fin (Fig S1). The unbent fish will be assumed symmetrical with respect to the x-z and x-y planes (up-down and left-right). The trailing edge of the caudal fin will be assumed straight. Unless explicitly indicated otherwise, the thickness of the fish (left-right) will be neglected. t, s and z_b will be the time, the semi-span of the fish projection onto the x-y plane, and the lateral displacement of the fish body from the swimming path in the x-z plane (Fig S1). The indices 'n', 't', '1' and '2' mark the anterior and posterior ends, the deepest section along the body, and the caudal peduncle. Where no confusion may result, the arguments t and x of all relevant functions will be tacitly omitted.

The lateral force per unit length acting on the body can be found from the rate of change of the linear momentum of the fluid in the plane perpendicular to the direction of swimming as the fish crosses this plane. The axial force per unit length can be found from by integrating the pressure. The reader is referred to Ref [2] for details. In general, the body is divided into three segments: (x_n, x_1), between the fish anterior end and the widest section; (x_1, x_2), between the widest section and the caudal peduncle; and (x_2, x_t), the caudal fin. Vortical wake is generated on the edges of the second segment, inducing side-wash on itself and on the third segment. Its effect is reflected in the following equations.

The force (per unit length) acting perpendicular to the plane of the body is

$$f_\perp = \pi \frac{D}{Dt}\left(s^2 v_\perp\right), \qquad x \in (x_n, x_1), \tag{S1}$$

$$f_\perp = \pi s^2 \frac{Dv_\perp}{Dt} = \pi \frac{D}{Dt}\left(s^2 v_\perp\right) - \pi v_\perp \frac{ds^2}{dx}, \qquad x \in (x_1, x_2), \tag{S2}$$

$$f_\perp = \pi \frac{D}{Dt}\left(s^2\left(v_\perp - v_{\perp\bullet}\right)\right) = \pi \frac{D}{Dt}\left(s^2 v_\perp\right) - \pi v_{\perp\bullet} \frac{ds^2}{dx}, \qquad x \in (x_2, x_t), \tag{S3}$$

where $D/Dt = \partial/\partial t + \partial/\partial x$ is the (reduced) convective derivative,

$$v_\perp = -\frac{Dz_b}{Dt}, \tag{S4}$$

$$v_{\perp\bullet}(t, x) = v_\perp\left(t - x + x_\bullet(x), x_*(x)\right), \tag{S5}$$

and $x_\bullet : (x_2, x_t) \to (x_1, x_2)$ is the location on the converging section of the fish for which $s(x_\bullet(x)) = s(x)$.[1] Adjusting notation, these are equations (14), (15), (16) and (12) in Ref [1].[2]

---

[1] Strictly speaking, $x_\bullet(x)$ exists for each $x \in (x_2, x_t)$ only if $s_t < s_1$. If $s_t > s_1$, $x_\bullet$ exists only on (x_2, x_1'), where x_1' is the solution of $s(x_1') = s_1$. On the rest of the caudal fin, (x_1', x_t), $v_{\perp\bullet}$ vanishes.

[2] v, lz_b and ls are equivalent to U, h and b ibid.

The force (per unit length) acting in the body plane is

$$f_{\parallel} = -\frac{\pi}{2} v_{\perp}^2 \frac{ds^2}{dx}, \qquad x \in (x_n, x_1) \tag{S6}$$

$$f_{\parallel} = 0 \qquad x \in (x_1, x_2), \tag{S7}$$

$$f_{\parallel} = -\frac{\pi}{2} (v_{\perp} - v_{\perp\bullet})^2 \frac{ds^2}{dx} \qquad x \in (x_2, x_t); \tag{S8}$$

These are equations (19)-(21) in Ref [1]. If no wake is generated on the second segment, $v_{\perp\bullet} = 0$ on $(-\infty, \infty) \times (x_1, x_t)$, and consequently, equations (S1) and (S6) apply for the entire fish, (x_n, x_t).

The rate of work done by the fish is

$$P = -\int_{x_n}^{x_t} f_{\perp} \frac{\partial z_b}{\partial t} dx, \tag{S9}$$

Separating the integration domain into the respective three segments, (x_n, x_1), (x_1, x_2), (x_2, x_t), and introducing (S1)-(S3), one will find

$$P = -\pi \int_{x_n}^{x_t} \frac{\partial z_b}{\partial t} \frac{D(s^2 v_{\perp})}{Dt} dx + \pi \int_{x_1}^{x_2} \frac{\partial z_b}{\partial t} v_{\perp} \frac{ds^2}{dx} dx + \pi \int_{x_2}^{x_t} \frac{\partial z_b}{\partial t} v_{\perp\bullet} \frac{ds^2}{dx} dx; \tag{S10}$$

whence

$$P = \frac{\pi}{2} \frac{\partial}{\partial t} \int_{x_n}^{x_t} \left(\left(\frac{\partial z_b}{\partial t} \right)^2 - \left(\frac{\partial z_b}{\partial x} \right)^2 \right) s^2 dx + \left(\pi s^2 \frac{\partial z_b}{\partial t} \frac{Dz_b}{Dt} \right)_{x = x_t}$$

$$-\pi \int_{x_1}^{x_2} \frac{\partial z_b}{\partial t} \frac{Dz_b}{Dt} \frac{ds^2}{dx} dx - \pi \int_{x_2}^{x_t} \frac{\partial z_b}{\partial t} \left(\frac{Dz_b}{Dt} \right)_{\bullet} \frac{ds^2}{dx} dx, \tag{S11}$$

by (S4). Noting that $s_n = s(x_n) = 0$ by assumption, the single non-trivial step in deriving (S11) was in regrouping the integrand in the first term on the right of (S10):

$$\frac{\partial z_b}{\partial t}\frac{D}{Dt}\left(s^2\frac{Dz_b}{Dt}\right)=\frac{D}{Dt}\left(s^2\frac{\partial z_b}{\partial t}\frac{Dz_b}{Dt}\right)-s^2\frac{Dz_b}{Dt}\frac{D}{Dt}\frac{\partial z_b}{\partial t}$$

$$=\frac{D}{Dt}\left(s^2\frac{\partial z_b}{\partial t}\frac{Dz_b}{Dt}\right)-\frac{1}{2}\frac{\partial}{\partial t}\left(s^2\left(\frac{Dz_b}{Dt}\right)^2\right)$$

$$=\frac{\partial}{\partial t}\left(s^2\frac{\partial z_b}{\partial t}\frac{Dz_b}{Dt}-\frac{1}{2}s^2\left(\frac{Dz_b}{Dt}\right)^2\right)+\frac{\partial}{\partial x}\left(s^2\frac{\partial z_b}{\partial t}\frac{Dz_b}{Dt}\right). \tag{S12}$$

Assuming that the motion is periodic, time-averaging of (S11) yields

$$\langle P\rangle=\pi s_t^2\left\langle\frac{\partial z_b}{\partial t}\frac{Dz_b}{Dt}\right\rangle_{x=x_t}-\pi\int_{x_1}^{x_2}\left\langle\frac{\partial z_b}{\partial t}\frac{Dz_b}{Dt}\right\rangle\frac{ds^2}{dx}dx-\pi\int_{x_2}^{x_t}\left\langle\frac{\partial z_b}{\partial t}\left(\frac{Dz_b}{Dt}\right)_{\bullet}\right\rangle\frac{ds^2}{dx}dx\,, \tag{S13}$$

where the angular brackets denote the period average of the respective quantity. The first term on the right of (S11) cancels out because for any periodic function g, $\langle \partial g/\partial t\rangle = 0$ by definition. Adjusting notation, equation (S13) is identical with equation (26) in Ref [1]. If no wake is generated on the second segment, or if the lateral motion of the second segment is vanishingly small, the second and the third terms fall out – see the paragraph following (S8) – leaving

$$\langle P\rangle=\pi s_t^2\left\langle\frac{\partial z_b}{\partial t}\frac{Dz_b}{Dt}\right\rangle_{x=x_t}, \tag{S14}$$

in accord with Ref [3].

The thrust of the fish is

$$T=\int_{x_n}^{x_t} f_{\perp}\frac{\partial z_b}{\partial x}dx-\int_{x_n}^{x_t} f_{\|}dx\,, \tag{S15}$$

where the last term reflects the leading edge suction on the forward segment of the fish and on the caudal fin. Introducing (S1)-(S3) and (S6)-(S8), it becomes

$$T=\pi\int_{x_n}^{x_t}\frac{D}{Dt}\left(s^2v_\perp\right)\frac{\partial z_b}{\partial x}dx-\pi\int_{x_1}^{x_2}v_\perp\frac{ds^2}{dx}\frac{\partial z_b}{\partial x}dx-\pi\int_{x_2}^{x_t}v_{\perp\bullet}\frac{ds^2}{dx}\frac{\partial z_b}{\partial x}dx$$

$$+\frac{\pi}{2}\int_{x_n}^{x_1}v_\perp^2\frac{ds^2}{dx}dx+\frac{\pi}{2}\int_{x_2}^{x_t}\left(v_\perp-v_{\perp\bullet}\right)^2\frac{ds^2}{dx}dx\,. \tag{S16}$$

Replacing the fourth term by the difference between the respective integrals on $\left(x_n,x_t\right)$, $\left(x_1,x_2\right)$ and $\left(x_2,x_t\right)$, and collecting the terms with the same integration limits, allows putting (S16) into the form:

$$T=\pi\int_{x_n}^{x_t}\left(\frac{D}{Dt}\left(s^2v_\perp\right)\frac{\partial z_b}{\partial x}+\frac{1}{2}v_\perp^2\frac{ds^2}{dx}\right)dx-\pi\int_{x_1}^{x_2}\left(\frac{\partial z_b}{\partial x}+\frac{1}{2}v_\perp\right)v_\perp\frac{ds^2}{dx}dx$$

$$+\frac{\pi}{2}\int_{x_2}^{x_t}\left(v_{\perp\bullet}-2\left(v_\perp+\frac{\partial z_b}{\partial x}\right)\right)v_{\perp\bullet}\frac{ds^2}{dx}dx\,. \tag{S17}$$

Introducing (S4) for $v_\perp$ yields

$$T=-\pi\frac{\partial}{\partial t}\int_{x_n}^{x_t}s^2\frac{\partial z_b}{\partial x}\frac{Dz_b}{Dt}dx+\left(\frac{\pi}{2}s^2\left(\left(\frac{\partial z_b}{\partial t}\right)^2-\left(\frac{\partial z_b}{\partial x}\right)^2\right)\right)_{x=x_t}$$

$$-\frac{\pi}{2}\int_{x_1}^{x_2}\left(\left(\frac{\partial z_b}{\partial t}\right)^2-\left(\frac{\partial z_b}{\partial x}\right)^2\right)\frac{ds^2}{dx}dx+\pi\int_{x_2}^{x_t}\left(\frac{1}{2}\left(\frac{Dz_b}{Dt}\right)_\bullet^2-\frac{\partial z_b}{\partial t}\left(\frac{Dz_b}{Dt}\right)_\bullet\right)\frac{ds^2}{dx}dx\,; \tag{S18}$$

recall that $s\left(x_n\right)=0$. The single non-trivial step in deriving (S18) was in recasting the integrand in the first term as

$$-\frac{D}{Dt}\left(s^2\frac{Dz_b}{Dt}\right)\frac{\partial z_b}{\partial x}+\frac{1}{2}\left(\frac{Dz_b}{Dt}\right)^2\frac{ds^2}{dx}=-\frac{D}{Dt}\left(s^2\frac{\partial z_b}{\partial x}\frac{Dz_b}{Dt}\right)+s^2\frac{Dz_b}{Dt}\frac{D}{Dt}\frac{\partial z_b}{\partial x}+\frac{1}{2}\left(\frac{Dz_b}{Dt}\right)^2\frac{ds^2}{dx}$$

$$=-\frac{D}{Dt}\left(s^2\frac{\partial z_b}{\partial x}\frac{Dz_b}{Dt}\right)+\frac{1}{2}\frac{\partial}{\partial x}\left(s^2\left(\frac{Dz_b}{Dt}\right)^2\right). \tag{S19}$$

Averaging (S18) over a single period yields

$$\langle T\rangle = \frac{\pi}{2}s_t^2\left\langle\left(\frac{\partial z_b}{\partial t}\right)^2 - \left(\frac{\partial z_b}{\partial x}\right)^2\right\rangle_{x=x_t} - \frac{\pi}{2}\int_{x_1}^{x_2}\left\langle\left(\frac{\partial z_b}{\partial t}\right)^2 - \left(\frac{\partial z_b}{\partial x}\right)^2\right\rangle\frac{ds^2}{dx}dx$$

$$+\pi\int_{x_2}^{x_t}\left\langle\frac{1}{2}\left(\frac{Dz_b}{Dt}\right)_\bullet^2 - \frac{\partial z_b}{\partial t}\left(\frac{Dz_b}{Dt}\right)_\bullet\right\rangle\frac{ds^2}{dx}dx\,; \tag{S20}$$

this is equation (25) in Ref [1]. As in (S13), if no wake is generated on the second segment, or if the lateral motion of the second segment is vanishingly small, the second and the third terms fall out, leaving

$$\langle T\rangle = \frac{\pi}{2}s_t^2\left\langle\left(\frac{\partial z_b}{\partial t}\right)^2 - \left(\frac{\partial z_b}{\partial x}\right)^2\right\rangle_{x=x_t}, \tag{S21}$$

in accord with Ref [3].

The particular case that will be needed for this study is the case of harmonic oscillations in which

$$z_b(t,x) = \mathrm{Re}\left(\hat{z}_b(x)e^{i\omega t}\right), \tag{S22}$$

$$\alpha_b(t,x) = \frac{\partial z_b(t,x)}{\partial x} = \mathrm{Re}\left(\hat{\alpha}_b(x)e^{i\omega t}\right), \tag{S23}$$

where the hat marks a complex amplitude. With these,

$$\left\langle\frac{\partial z_b}{\partial t}\frac{Dz_b}{Dt}\right\rangle = \frac{1}{2}\omega\left(\omega|\hat{z}_b|^2 + \mathrm{Im}\left(\tilde{z}_b\hat{\alpha}_b\right)\right), \tag{S24}$$

$$\left\langle\frac{\partial z_b}{\partial t}\left(\frac{Dz_b}{Dt}\right)_\bullet\right\rangle = \frac{1}{2}\mathrm{Re}\left(\left(\omega^2\tilde{z}_b\hat{z}_\bullet - i\omega\tilde{z}_b\hat{\alpha}_\bullet\right)e^{-i\omega(x-x_\bullet)}\right), \tag{S25}$$

$$\left\langle\left(\frac{\partial z_b}{\partial t}\right)^2 - \left(\frac{\partial z_b}{\partial x}\right)^2\right\rangle = \frac{1}{2}\left(\omega^2|\hat{z}_b|^2 - |\hat{\alpha}_b|^2\right), \tag{S26}$$

$$\left\langle\left(\frac{Dz_b}{Dt}\right)_\bullet^2\right\rangle = \frac{1}{2}\left|i\omega\hat{z}_\bullet + \hat{\alpha}_\bullet\right|^2 \tag{S27}$$

by (S5). In these, the tilde marks a complex conjugate, whereas $\hat{z}_\bullet = \hat{z}_b(x_\bullet)$, $\hat{\alpha}_\bullet = \hat{\alpha}_b(x_\bullet)$. Thus,

$$\langle P\rangle = \frac{\pi}{2}s_t^2\,\mathrm{Re}\Big(\omega^2\left|\hat{z}_t\right|^2 - i\omega\tilde{z}_t\hat{\alpha}_t\Big) - \frac{\pi}{2}\mathrm{Re}\int_{x_1}^{x_2}\Big(\omega^2\left|\hat{z}_b\right|^2 - i\omega\tilde{z}_b\hat{\alpha}_b\Big)\frac{ds^2}{dx}dx$$

$$-\frac{\pi}{2}\mathrm{Re}\int_{x_2}^{x_t}\tilde{z}_b\left(\omega^2\hat{z}_\bullet - i\omega\hat{\alpha}_\bullet\right)e^{-i\omega(x-x_\bullet)}\frac{ds^2}{dx}dx\,, \tag{S28}$$

$$\langle T\rangle = \frac{\pi}{4}s_t^2\left(\omega^2\left|\hat{z}_t\right|^2 - \left|\hat{\alpha}_t\right|^2\right) - \frac{\pi}{4}\int_{x_1}^{x_2}\left(\omega^2\left|\hat{z}_b\right|^2 - \left|\hat{\alpha}_b\right|^2\right)\frac{ds^2}{dx}dx$$

$$+\frac{\pi}{4}\int_{x_2}^{x_t}\left|i\omega\hat{z}_\bullet + \hat{\alpha}_\bullet\right|^2\frac{ds^2}{dx}dx - \frac{\pi}{2}\mathrm{Re}\int_{x_2}^{x_t}\tilde{z}_b\left(\omega^2\hat{z}_\bullet - i\omega\hat{\alpha}_\bullet\right)e^{-i\omega(x-x_\bullet)}\frac{ds^2}{dx}dx\,; \tag{S29}$$

where the tilde marks a complex conjugate. The propulsion efficiency can be defined as

$$\eta = \langle T\rangle / \langle P\rangle\,, \tag{S30}$$

recall that in dimensionless representation the swimming velocity is unity. The resulting expression is unwieldy, and hence will not be presented in explicit form.

Equations (S28)-(S29) can be simplified when the converging, the wake releasing, segment of the fish is sufficiently short (and $s_1 \geq s_t$). In this case, one can set $x_\bullet \approx x_1$, $\hat{z}_\bullet \approx \hat{z}_1$, and $\hat{\alpha}_\bullet \approx \hat{\alpha}_1$, with which

$$\langle P\rangle = \frac{\pi}{2}s_t^2\,\mathrm{Re}\Big(\omega^2\left|\hat{z}_t\right|^2 - i\omega\tilde{z}_t\hat{\alpha}_t\Big) - \frac{\pi}{2}\mathrm{Re}\Big(\omega^2\left|\hat{z}_1\right|^2 - i\omega\tilde{z}_1\hat{\alpha}_1\Big)\left(s_2^2 - s_1^2\right)$$

$$-\frac{\pi}{2}\mathrm{Re}\left(\left(\omega^2\hat{z}_1 - i\omega\hat{\alpha}_1\right)e^{i\omega x_1}\int_{x_2}^{x_t}\tilde{z}_b e^{-i\omega x}\frac{ds^2}{dx}dx\right), \tag{S31}$$

$$\langle T\rangle = \frac{\pi}{4}s_t^2\left(\omega^2\left|\hat{z}_t\right|^2 - \left|\hat{\alpha}_t\right|^2\right) - \frac{\pi}{4}\Big(\omega^2\left|\hat{z}_1\right|^2 - \left|\hat{\alpha}_1\right|^2\Big)\left(s_2^2 - s_1^2\right)$$

$$+\frac{\pi}{4}\left|i\omega\hat{z}_1 + \hat{\alpha}_1\right|^2\left(s_t^2 - s_2^2\right) - \frac{\pi}{2}\mathrm{Re}\left(\left(\omega^2\hat{z}_1 - i\omega\hat{\alpha}_1\right)e^{i\omega x_1}\int_{x_2}^{x_t}\tilde{z}_b e^{-i\omega x}\frac{ds^2}{dx}dx\right). \tag{S32}$$

If, in addition, the amplitudes $\hat{z}_1$ and $\hat{\alpha}_1$ of the deepest section of the fish are small as compared with $\hat{z}_t$ and $\hat{\alpha}_t$, these reduce to

$$\langle P \rangle = \frac{\pi}{2} s_t^2 \,\mathrm{Re}\left(\omega^2 \left|\hat{z}_t\right|^2 - i\omega \tilde{z}_t \hat{\alpha}_t\right) + \ldots = \frac{\pi}{2} s_t^2 \left(\omega^2 \left|\hat{z}_t\right|^2 + \omega \,\mathrm{Im}\left(\tilde{z}_t \hat{\alpha}_t\right)\right) + \ldots, \tag{S33}$$

$$\langle T \rangle = \frac{\pi}{4} s_t^2 \left(\omega^2 \left|\hat{z}_t\right|^2 - \left|\hat{\alpha}_t\right|^2\right) + \ldots, \tag{S34}$$

where the ellipses stand for the terms of the order of $\omega^2 \left|\tilde{z}_t \hat{z}_1\right|$ and $\omega \left|\tilde{z}_1 \hat{\alpha}_1\right|$. Concurrently,

$$\eta = \frac{1}{2} \frac{\omega^2 \hat{z}_t \tilde{z}_t - \hat{\alpha}_t \tilde{\alpha}_t}{\omega^2 \hat{z}_t \tilde{z}_t + \omega \,\mathrm{Im}\left(\tilde{z}_t \hat{\alpha}_t\right)} + \ldots. \tag{S35}$$

Effectively, equations (S33)-(S35) manifest the leading order approximation for all those cases where the wake shed from the converging part of the fish is weak. They are exact in those cases where the wake is nonexistent (see the paragraph following (S8)).

When $\hat{\alpha}_t$ and $\hat{z}_t$ are in phase, $\mathrm{Im}\left(\tilde{z}_t \hat{\alpha}_t\right)$ vanishes, leaving the propulsion efficiency

$$\eta = \frac{1}{2} \left(1 - \frac{\left|\hat{\alpha}_t\right|^2}{\omega^2 \left|\hat{z}_t\right|^2}\right) \tag{S36}$$

to be less than $1/2$. The efficiency can be increased by making $\mathrm{Im}\left(\tilde{z}_t \hat{\alpha}_t\right)$ negative and large compared with $\left|\hat{\alpha}_t\right|^2$. It can happen if $\hat{\alpha}_t$ lags 90 degrees behind $\hat{z}_t$. This implies turning the caudal end left when it moves right, and vice versa.

References

[1] Yates G.T., "Hydromechanics of body and caudal fin propulsion," in Weihs D. and Webb P.W., *Fish biomechanics*, Praeger, 1983, pp. 207-213

[2] Neumann J.N. and Wu T.Y., "A generalized slender-body theory for fish-like forms," *Journal of Fluid Mechanics* **57**, 1973, pp. 673-693

[3] Lighthill J., "Note on the swimming of slender fish," *Journal of Fluid Mechanics* **9**, 1960, pp. 305-317
