# Supplementary material for: Hydrodynamics of a Flexible Soft-Rayed Caudal Fin
Source: PLoS One. 2016 Oct 3;11(10):e0163517. doi: 10.1371/journal.pone.0163517 (PMC5047476; doi:10.1371/journal.pone.0163517)
Supplement: S2 File — (PDF) [file pone.0163517.s002.pdf]

# Drag coefficient of a fish

Hydrodynamic drag is commonly expressed as

$$D = (1/2)\,\rho S v^2 C_D, \tag{S1}$$

where $\rho$ is the density of water, $v$ is the swimming speed, $S$ is an arbitrary reference area (it was chosen as the maximal cross section area of the body in the text), and $C_D$ is the respective drag coefficient. The aim of this document is to furnish a crude estimate for the drag coefficient of a fish. It will be tacitly assumed that the fish generates no lift.

Drag is contributed by the body of the fish (it will be marked by the index '0') and its $N$ fins (they will be marked by the indices '1',…,'$N$'). Based on equations (12.24) and (12.27) of Ref [1], the drag coefficient can be expressed as

$$C_D = \sum_{n=0}^{N} \frac{S_n}{S} C_f(\mathrm{Re}_n) F_n I_{n0}, \tag{S2}$$

where $S_0$, …, $S_N$ are the wet areas of the respective constituents; $F_0$, …, $F_N$ are empirical corrections accounting for increase in drag due to flow separation; $I_{00},\ldots,I_{N0}$ are empirical corrections accounting for an increase in drag due to hydrodynamic interaction with the body of the fish;

$$\mathrm{Re}_n = \rho v l_n / \mu, \tag{S3}$$

is the Reynolds number based on the stream-wise dimension of the respective constituent, $l_n$; and, finally,

$$C_f(\mathrm{Re}) = 0.455 / (\log_{10} \mathrm{Re})^{2.58} \tag{S4}$$

is the effective friction coefficient. In (S3), $\mu$ is the viscosity of water. Equation (S4) is based on a tacit assumption that the boundary layer is turbulent.

Approximating the body by a double-ogive of length $l_0$ and maximal diameter $d_0$,

$$S_0 = (2/3)\pi l_0 d_0 . \tag{S5}$$

Based on equation (12.31) of Ref [1],

$$F_0 = 1 + 60(d_0/l_0)^3 + 0.0025(l_0/d_0) . \tag{S6}$$

Because there is no hydrodynamic interaction between the body and itself, $I_{00}$ should have been unity. Nonetheless, we set $I_{00} = 1.1$ to account, at least partially, for the drag of the gills.

$S_1, \ldots, S_N$ are, approximately, twice the projected areas of the respective fins. Based on equation (12.30) of Ref [1],

$$F_n \approx 1 + 2(t_n/l_n) + 100(t_n/l_n)^3 , \tag{S7}$$

where $t_1, \ldots, t_N$ are the thicknesses of the fins. $I_{n0} = 1.4$ is set for every $n > 0$ based on the suggestion appearing on page 283 ibid.

Choosing the reference area $S$ as the maximal cross section area of the body, the contribution of the body, $(S_0/S) C_f(\mathrm{Re}_0) F_0 I_{00}$, is shown in Fig S1a as a function of the respective Reynolds number and the ratio $l_0/d_0$. The contribution $C_f(\mathrm{Re}_n) F_n I_{n0}$ of the *n*th fin is shown on Fig 1b.

For example, consider a 1 m fish, 0.18 m across, moving at 1 body length per second in 25°C water. The fish has a few similar fins with thickness-to-chord ratio of 0.1 and 0.05 m chord, their combined area that is twice the cross section area of the body. The Reynolds number, based on the body length, is $10^6$, by (S3). From Fig S1a, $(S_0/S) C_f(\mathrm{Re}_0) F_0 I_{00} \approx (1) \cdot 0.1 = 0.1$. The Reynolds number, based on the fin chord, is 50,000. From Fig S2b, $\sum_{n=1}^{N} (S_n/S) C_f(\mathrm{Re}_n) F_n I_{n0} \approx (2) \cdot 0.014 = 0.028$. The drag coefficient of the fish, based on its cross section area, is the sum of the two, approximately, 0.13.

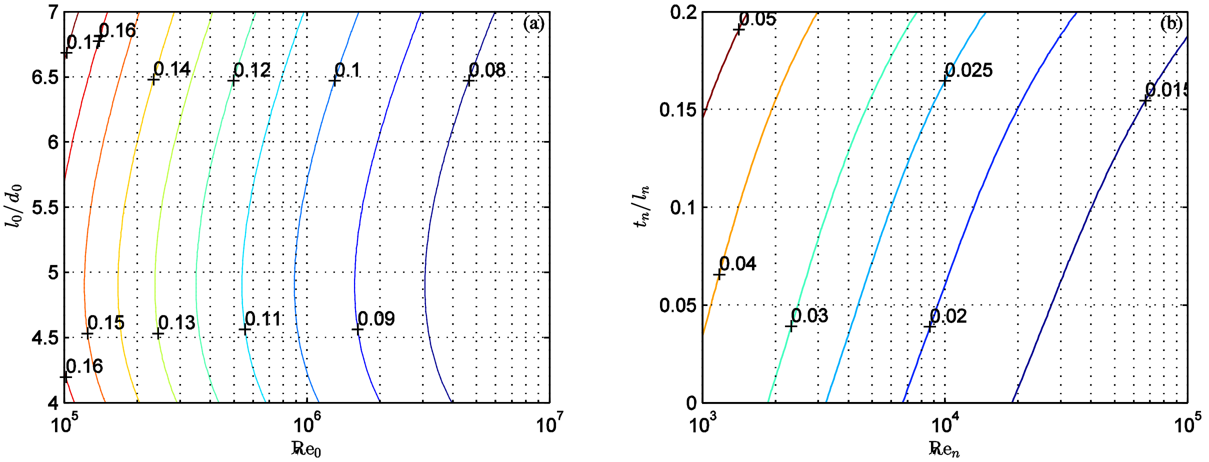


**Fig S1**: Contours of constant drag coefficient over the map of a shape parameter and the Reynolds number. Drag coefficient of the body (based on its cross section area) is shown on the left; the shape parameter is the length-to-diameter ratio. Drag coefficient of a fin (based on its wet area) is shown on the right; the shape parameter is the thickness-to-chord ratio.

## References


[1] Raymer D.P., *Aircraft design: a conceptual approach*, AIAA educational series, Washington DC, 1992, pp 279-281
